# 危重及难治性免疫检查点抑制剂相关毒性反应诊治建议及探索

**DOI:** 10.3779/j.issn.1009-3419.2019.10.01

**Published:** 2019-10-20

**Authors:** 汉萍 王, 鹏 宋, 晓燕 斯, 潇潇 郭, 玥 李, 佳鑫 周, 炼 段, 丽 张, 孟昭 王, 力 张

**Affiliations:** 1 100730 北京，中国医学科学院，北京协和医院呼吸内科 Department of Respiratory Medicine, Peking Union Medical College Hospital, Peking Union Medical College and Chinese Academy of Medical Sciences, Beijing 100730, China; 2 100730 北京，中国医学科学院，北京协和医院心内科 Department of Cardiology, Peking Union Medical College Hospital, Peking Union Medical College and Chinese Academy of Medical Sciences, Beijing 100730, China; 3 100730 北京，中国医学科学院，北京协和医院消化内科 Department of Gastroenterology, Peking Union Medical College Hospital, Peking Union Medical College and Chinese Academy of Medical Sciences, Beijing 100730, China; 4 100730 北京，中国医学科学院，北京协和医院风湿免疫科 Department of Rheumatology, Peking Union Medical College Hospital, Peking Union Medical College and Chinese Academy of Medical Sciences, Beijing 100730, China; 5 100730 北京，中国医学科学院，北京协和医院内分泌科 Department of Endocrinology, Peking Union Medical College Hospital, Peking Union Medical College and Chinese Academy of Medical Sciences, Beijing 100730, China; 6 100730 北京，中国医学科学院，北京协和医院检验科 Department of CilincalLaboratory, Peking Union Medical College Hospital, Peking Union Medical College and Chinese Academy of Medical Sciences, Beijing 100730, China

**Keywords:** 免疫检查点抑制剂, 免疫相关不良反应, 难治性, 危重性, 降阶梯治疗, Immune checkpoint inhibitor, Immunotherapy-related toxicities, Refractory, Severe, De-escalation Therapy

## Abstract

免疫检查点抑制剂（immune checkpoint inhibitors, ICIs）的应用改写了很多恶性肿瘤的治疗策略，成为肿瘤治疗的又一个里程碑。ICIs作用原理可以理解为“刹车理论”，在“松开刹车”后会带来一系列的全身性毒副反应，其中部分可能为危重和难治性，甚至具有潜在致死性。本文对免疫相关不良事件（immune-related adverse effects, irAEs）相关的最新国内外指南及共识中关于3度-4度irAEs的诊治建议进行了汇总，包括欧洲肿瘤内科学会年会（European Society for Medical Oncology, ESMO）、美国国立综合癌症网络（National Comprehensive Cancer Network, NCCN）/美国临床肿瘤学会（American Society of Clinical Oncology, ASCO）、肿瘤免疫治疗学会（Society for Immunotherapy of Cancer, SITC）和中国临床肿瘤学会（Chinese Society of Clinical Oncology, CSCO）指南，并复习了2019年5月20日前公开发表的关于irAEs的个案报告和相关的综述文献后，对以上指南和共识中尚未纳入的3级-4级irAEs的诊治建议进行补充，重点介绍了特异性免疫抑制药物在不同的irAEs中成功应用的情况，包括抗白介素6（interleukin 6, IL-6）阻断、抗CD20单抗、抗肿瘤坏死因子α（tumor necrosis factor-α, TNFα）单抗、抗整合素4单抗、Janus激酶抑制剂、血小板生成素受体激动剂和抗胸腺细胞球蛋白（antithymocyte globulin, ATG）。本文对于类固醇激素在irAEs中超大剂量使用和升级使用及反复使用提出质疑，并强调应同时关注激素继发的感染、肿瘤进展和无法满足ICIs再挑战的等问题。本文提出对于危重和难治性irAEs的“降阶梯治疗”原则，建议应尽早使用细胞因子靶向药物这些特异性免疫抑制药物。免疫治疗时代诸多的irAEs是传统化疗及小分子靶向治疗时代不曾有过的，不断地挑战肿瘤科大夫的知识储备和临床基本技能。因此，建立肿瘤多学科讨论体系对于肿瘤患者的治疗管理极为重要。

## Abstract (en)

The application of immunological checkpoint inhibitors (ICIs) has modified many treatment strategies of malignant tumors, which has become a milestone in cancer therapy. The principle of action can be explained as "brake theory". After releasing the brakes by ICIs, unprecedented systemic toxicities, even some refractory and fatal immune-related adverse effects (irAEs) may develop. In this article, we summarized the recommended treatments of grade 3-4 severe irAEs in the latest European Society for Medical Oncology (ESMO), National Comprehensive Cancer Network (NCCN)/American Society of Clinical Oncology (ASCO), Society for Immunotherapy of Cancer (SITC) and Chinese Society of Clinical Oncology (CSCO) guidelines and consensus. We also performed a systemic review of case reports and reviews of irAEs up to May 20, 2019 in PubMed and Chinese journals. Successful applications of specific immunosuppressive drugs and stimulating factors beyond the above guidelines and consensus were supplemented and highlighted, including agents blocking interleukin 6 (IL-6), rituximab, anti-tumor necrosis factor-α (TNFα) monoclonal antibody (mAb), anti-integrin 4 mAb, Janus kinase inhibitors, thrombopoietin receptor agonists and antithymocyte globulin (ATG) *etc*. We put some concerns of using high-dose steroids for long-term, and emphasize the secondary infections, tumor progression, and unavailability of ICI re-challenge during steroid treatment. We propose the "De-escalation Therapy" principle for severe and refractory irAEs, and suggest that immunosuppressive drugs specifically targeting cytokines should be used as early as possible. Many irAEs in the era of immunotherapy are unprecedented compared with traditional chemotherapy and small-molecule targeted therapy, which is a big challenge to oncologists. Therefore, the establishment of multidisciplinary system is very important for the management of cancer patients.

## 1 背景

肿瘤的免疫治疗最早可追溯至1863年科利毒素的应用，至今经历了卡介苗（bacillus calmette-guerin, BCG）、干扰素α（interferon-α, IFNα）、白介素-2（interleukin-2, IL-2）、主要组织相容性复合体（major histocompatibility complex, MHC）、肿瘤坏死因子（tumor necrosis factor, TNF）等^[1, 2]^，直到最近出现了免疫检查点抑制剂（immune checkpoint inhibitors, ICIs）。与传统的化疗和靶向治疗不同，ICI不再以肿瘤细胞为靶点直接杀伤肿瘤细胞，而是以免疫细胞为靶点，通过增强抗肿瘤免疫反应起作用，为晚期肿瘤患者带来持久的临床获益，目前主要包括程序性死亡受体1（programmed cell death protein 1, PD-1）/程序性死亡受体配体1（programmed cell death protein ligand 1, PD-L1）抑制剂和细胞毒性T淋巴细胞相关抗原4（cytotoxic T lymphocyte associated antigen-4, CTLA-4）抑制剂。

然而，PD-1/PD-L1抑制剂和CTLA-4抑制剂为晚期肿瘤患者带来长期持续的临床获益的同时，也可能引起全身系统性的毒副反应（immune-related adverse effects, irAEs），甚至危及生命。轻度irAEs（1级-2级）和大部分3级-4级的irAEs经过早期类固醇激素的治疗后可控制良好，部分患者可再次接受ICI治疗，但仍有一小部分irAEs临床表现严重或不能通过类固醇有效控制，属于危重或难治类型，患者后续可能因irAEs未控制、类固醇使用继发的不良反应或原发肿瘤进展等原因危及生命。而其中双免疫检查点抑制剂联合治疗时出现危重irAEs的概率更高。

迄今为止，国内外已经有一系列关于irAE管理的指南和共识可供使用，包括欧洲医学肿瘤学协会（European Society of Medical Oncology, ESMO）^[3]^、美国国家综合癌症网络（National Comprehensive Cancer Network, NCCN）^[4]^、国际肿瘤免疫治疗毒性反应管理协作协会（Society for Immunotherapy of Cancer Toxicity Management Working Group）^[5]^和中国临床肿瘤学会（Chinese Society of Clinical Oncology, CSCO）均有高质量的irAEs管理指南发布^[6]^。这些指南对于常见irAEs的处理提出了详细建议，也强调早期识别与处理，强调了鉴别诊断（如感染等并发症、肿瘤进展和基础疾病的存在及活动状态）等，但是对于危重及难治性irAEs给予的指导意见较少且笼统。并且指南并没有将“难治性irAEs”这一临床确实存在的情况作为一种特殊的irAEs进行阐述和推荐，而临床上我们只有克服“难治性irAEs”才能确实提高irAEs的管理成功率。

各大指南对严重的irAEs（3级-4级）的处理原则目前大致类似，即遵循CTCAE-4.03版的处理原则^[7]^，3级-4级irAEs患者应住院或住重症监护病房（intensive care unit, ICU）治疗；接受全身类固醇治疗；对类固醇治疗3 d-5 d后症状未能缓解的患者，可在专科医师指导下进一步治疗；应暂时或永久停用ICIs，4级毒性永久停用ICIs。全身类固醇治疗方面，建议静脉使用甲基泼尼松龙1 mg/kg/d-2 mg/kg/d，连续3 d，若症状缓解逐渐减量至1 mg/kg/d维持4周-6周，后逐步减量，至6周左右停药。但是关于类固醇激素的具体种类、剂量、剂型方面没有更多推荐，对于激素继发的不良反应方面没有深刻描述，对于激素不敏感者的后续治疗没有进一步的推荐。因此对于这些危重和难治的irAEs仍是临床面临的主要问题。本文针对近年来危重及难治irAE在临床应用中存在的问题及其解决策略的相关研究进展进行了总结，旨在为基础与临床肿瘤研究人员解决面临的问题提供参考依据。

## 2 方法及依据

本文对于现有指南、共识及文献进行了深入评估和总结，具体包括：（1）对五大指南和共识中3-4级毒性的建议进行了总结，只要其中一个指南和共识给出建议的均包括在内，进行统一介绍，特殊情况指出各个指南共识中的意见分歧和不统一。（2）完成了对Pubmed、Embase数据库检索到的（时间截止到2019年5月20日，检索nivolumab、pembrolizumab、atezolizumab、avelumab、durvalumab、ipilimumab、tremelimumab或联合使用；immune-related adverse event或immune-mediated AEs或immune-mediated toxicity；English；筛查case）个案报道共232例进行了逐个复习及总结，对相关综述和文献进行了复习，补充了指南和共识中尚未提及的irAEs的类型名称，尤其是其中的危重型irAEs进行指南补充。（3）对irAEs治疗可能相关的几类免疫抑制药物进行推荐，介绍协定处方的用法。

## 3 指南和共识中各系统推荐总结和建议描述及本文的建议

以下按照CSCO指南的irAE攥写顺序进行介绍，分成指南和共识推荐，及本文建议两部分，主要针对危重型irAE的名称和处理策略进行介绍，同时指出目前指南和共识中尚未提到的irAE种类及建议。

### 3.1 皮肤系统

根据现有指南/共识，斑丘疹/皮疹、瘙痒及反应性皮肤毛细血管增生症（reactive skin capillary hyperplasia, CCEP）分为3个级别，无4级毒性，未考虑难治性AE的情况。其中CCEP仅在CSCO指南中见到。危重型皮疹定义为大疱性皮炎、Stevens-Johnson综合征（Stevens-Johnson syndrome, SJS）即中毒性表皮坏死松解症（SJS/toxic epidermal necrolysis, SJS/TEN）和伴嗜酸粒细胞增多和系统症状的药疹（drug rash with eosinophilia and systemic symptoms, DRESS），多为3级-4级毒性，并可同时出现。处理策略包括永久停用ICIs，泼尼松/甲基泼尼松龙1 mg/kg/d-2 mg/kg/d，需要住院治疗。

我们认为，在真实世界中已经可见到的皮肤改变如银屑病、血管炎伴紫癜性皮疹等均可呈危重和难治性^[8]^。治疗可考虑泼尼松，研究和病例报道显示硫唑嘌呤、霉酚酸酯、甲氨蝶呤、四环素类抗生素（四环素、多西环素、米诺环素等）、达普松和烟酰胺可以作为有效的类固醇替代药物。一项包含21例银屑病患者的回顾性研究指出ICIs诱发银屑病在男性中高发18/21（85.7%）；出现银屑病后，2例（9.5%）全身激素治疗，1例（4.8%）依曲替酸治疗，1例（4.8%）光疗；19例（90.5%）治疗后缓解。对于急性期严重银屑病，治疗上可以考虑应用抗TNFα、IL-1阻断剂、抗IL-23和抗IL-12单抗，皮科严重银屑病治疗的策略中合并有关节疼痛时考虑使用类固醇激素。当出现对抗TNFα不敏感的难治性银屑病时，IL-1阻断剂、抗IL-23和抗IL-12单抗已经有成功的报道。皮肤血管炎的治疗推荐用抗CD20单抗^[9]^。

### 3.2 内分泌系统

指南/共识中描述的内分泌系统毒性包括甲状腺、肾上腺、垂体和胰腺等炎症以及胰腺受累引发的高血糖。均未推荐类固醇激素的使用，4级毒性均未涉及停药，激素替代和对症处理恢复I度后均可以再次挑战ICIs治疗。

胰岛损伤者无论是否应用激素治疗其胰岛功能均无法完全恢复，患者需终生使用胰岛素控制血糖。目前认为部分患者性腺、甲状腺功能有可能恢复，但肾上腺功能无法恢复，需终生替代治疗。其中ipilimumab治疗者需高度警惕垂体炎。对于内分泌系统的这些损伤，目前指南/共识仅建议补充相应的激素。另外，临床上还见到腮腺炎和唾液腺炎的患者，目前尚无相应的处理意见。

### 3.3 消化系统

消化系统毒性包括肝脏毒性、胰腺毒性（急性胰腺炎）、胃肠毒性（腹泻/结肠炎）、食道炎、胃炎、十二指肠炎、小肠炎、胆管炎和胰管炎。

#### 3.3.1 肝脏毒性

各指南/共识对于肝毒性的分级标准不一，ESMO和NCCN/ASCO指南未将胆红素升高纳入诊断分级标准之内；肿瘤免疫治疗学会（Society for Immunotherapy of Cancer, SITC）的4级毒性标准较低；CSCO相对全面。只有CSCO指南要求在irAE处理过程中，病情缓解不满意时进行病毒性肝炎的检查（HBC和HCV），这可能与中国国情有关。总体的治疗原则是，静脉使用类固醇激素、麦考酚酯和他克莫司相继使用。疗效不佳时，观察窗口期为2 d-3 d。建议专科治疗。抗胸腺细胞球蛋白（antithymocyte globulin, ATG）作为激素无效时的推荐治疗，不推荐英夫利昔单抗（大多以风险过大提出警戒）。建议永久停用免疫治疗。在同一类ICIs中不提倡换用，在不同类ICI中可以慎重考虑，如CTLA-4单抗更换成PD-1及PD-L1单抗。

我们建议应重视谷草转氨酶（aspartate aminotransferase, AST）/谷丙转氨酶（alanine aminotransferase, ALT）比值的变化，这一指标可能反映是否存在肝脏以外的损伤；同时应注意胆红素与肝酶不同步升高现象的临床意义。胆酶分离往往提示肝功能极度恶化、危及生命的紧急情况，但是在irAE的范畴内是否具有相同的临床意义尚不清楚，有待较多探索。另外，在危重和难治的肝毒性处理中已经有应用IL-6单抗、抗CD20、抗TNFα单抗成功的案例，最佳治疗尚有待进一步探索^[9]^。

#### 3.3.2 胰腺毒性（急性胰腺炎）

只有NCCN/ASCO的指南中将胰腺毒性作为一项独立的毒性反应列出，建议2级即停止ICI并予以类固醇激素治疗。3级-4级胰腺毒性定义为淀粉酶/脂肪酶升高至 > 3倍正常值上限，和/或放射学异常，和/或中度腹痛或呕吐，及血液动力学不稳定，还应注意血糖变化。治疗建议类固醇激素及专科治疗，效差可考虑麦考酚酯。而其他指南未提及此毒性及治疗建议。

我们认为，由于目前报道的病例数少，治疗经验不足，关于类固醇激素和麦考酚酯以外的其他治疗尚无相应报告和推荐，但从irAE的发病机理来看，特异性免疫抑制治疗值得进一步探索。

#### 3.3.3 胃肠毒性

胃肠毒性主要表现腹泻/结肠炎，根据CSCO的意见，2级毒性即建议类固醇激素治疗，无效时即推荐加用英夫利昔单抗；3度-4度腹泻可以无需等待肠镜检查即可开始类固醇激素，如48 h激素无效时考虑加用英夫利昔单抗；如果仍无效，可考虑α4β7整合素拮抗剂维多珠单抗治疗，少有建议麦考酚酯。ESMO的指南不排斥止泻药的应用，其他指南对止泻药未做相关建议。

本文建议对于初发初治、临床典型的3度-4度腹泻可以无需等待肠镜检查即开始类固醇激素。但是对于长期使用类固醇激素效差的难治性irAE需要尽快进行肠镜检查，首先排除感染，如巨细胞病毒（cytomegalovirus, CMV）和难辨梭杆菌等继发感染，并且在长期反复腹泻的病例应该对比肠镜检查结果。对于难治性肠炎腹泻尤其抗TNFα单抗效果不佳时还可以尝试使用IL-6抑制剂、IL-1抑制剂、IL-17单抗、IL-23单抗及IL-12单抗^[9]^。必要时需外科手术引流术和穿孔修复术^[10]^。另外，消化道毒性方面也有关于食道炎、胃炎、十二指肠炎和小肠炎、胆管炎及胰管炎的个案报道。

### 3.4 肺毒性

#### 3.4.1 肺间质损伤

肺间质损伤中急性间质性肺炎或弥漫性肺泡损伤综合征（diffuse alveolar damage syndrome, DADS）是其中最危重可危及生命的事件。其他如机化性肺炎（organising inflammatory pneumonia）和肉瘤样肺肉芽肿病等肺部病变，病情相对缓和，但有时与感染或疾病进展鉴别困难。对于3级及以上肺炎，推荐收入院治疗，经验性使用广谱抗生素，予以大剂量静脉类固醇激素治疗（如（甲基）泼尼松龙1 mg/kg/d-4 mg/kg/d或等效药物），10%-15%的患者对激素治疗可能不敏感，建议对皮质类固醇治疗2 d无好转的患者加用免疫抑制剂治疗，可选择英夫利西单抗、麦考酚酸或环磷酰胺。指南均建议类固醇激素的减量应该非常缓慢谨慎，应该超过4周-8周逐渐停药。建议可静脉注射免疫球蛋白，建议ICU的支持，支气管镜检查指导治疗应该成为常规，可以纠正经验治疗的偏差。

本文建议，虽然肺间质损伤的死亡率很高，但死亡原因除了irAE难以控制外，更有一大部分患者因继发了不能控制的感染死亡，因此我们认为未来对于类固醇激素的大量长期应用应该加以避免，深入了解细胞因子拮抗剂等药物、与激素合理配伍早期应用或可成为可能，如IL-6抑制剂等^[9]^。另外文献^[11]^也有报道ICI治疗后出现Good-pasture病的个案，临床应该注意鉴别。

#### 3.4.2 肺结节病

肺结节病无危重症描述。只有CSCO指南指出肺外（眼、心肌、神经系统和肾脏）结节病及结节病伴发高钙血症者属重症范畴，类固醇激素的推荐剂量1 mg/kg-2 mg/kg，2周-4周内逐渐停药，属减量和停药较快的类型，SITC指南则认为这样的处理欠妥。如临床考虑应用英夫利昔单抗情况，则应先除外结核。

本文建议，肺结节的病理学诊断至关重要，非常容易与假性进展、感染等相混淆。结节病样的改变不仅可出现在肺部和淋巴结，也有报道出现在腹腔淋巴结和脑实质中^[12]^。对于与整体病情不符的新发病灶应该通过组织活检予以明确。

### 3.5 类风湿性关节/骨骼/肌肉毒性

#### 3.5.1 类风湿性关节毒性

类风湿性关节炎样毒性分级最高为3级，建议暂停ICIs，使用4周-6周泼尼松（1 mg/kg/d），如果2周内症状无改善请专科治疗，考虑其他免疫抑制剂（如甲氨喋呤、柳氮磺胺嘧啶或来氟米特等）。甲氨喋呤（初始计量为15 mg/周，同时每天补充叶酸，滴定至最大剂量）25 mg/周。对类固醇激素和抗炎治疗效果不佳的关节炎，考虑使用IL-6抑制剂托珠单抗或英夫利昔单抗。而NCCN/ASCO指南则在激素适量应用疗效不佳时推荐首选托珠单抗或英夫利昔单抗治疗。关节炎症状也可持续2年以上并需要使用免疫调节剂药物，也可能与其他的irAE同时出现（如：干燥综合征、腮腺炎、炎症性肌炎、血管炎和风湿性多肌痛、系统性红斑狼疮和结节病）。

本文建议，在真实世界中存在关于严重或难治性关节炎的报道，使用IL-1抑制剂、IL-6抑制剂、抗IL-17单抗、抗TNFα单抗、抗IL-23和抗IL-12单抗及Janus激酶抑制剂均可有效控制，并可缩短整体用药周期^[9]^。

#### 3.5.2 肌炎或肌痛

中重度肌炎需停止ICIs，收入院，立即用1 mg/kg/d-2 mg/kg/d甲基泼尼松龙治疗。对于重症和难治性的案例应考虑肌肉活检，连续监测肌酸激酶/醛缩酶水平，考虑静脉免疫球蛋白治疗及考虑血浆置换，直至症状消失或停用类固醇。未推荐免疫抑制剂（包括特异性和非特异性）。

我们认为，关于肌炎各大指南/共识均未推荐免疫抑制剂治疗，但有合并肌炎和其他系统损伤时应用特异性免疫抑制剂（细胞因子拮抗剂）治疗时一同好转的报告^[13]^。部分肌炎与心肌炎合并出现，部分肌炎虽不合并心肌炎但可累及吞咽肌、呼吸肌等影响呼吸吞咽功能，部分肌炎后期可导致重症肌无力样改变且恢复缓慢，期间患者容易合并肺部感染等导致死亡。因此我们认为，对于肌肉受累广泛的重度肌炎，合并心肌等其他脏器受累，或影响吞咽或呼吸肌的重症肌炎，可探索早期使用类固醇激素联合免疫制剂的治疗方案，以尽快控制肌肉炎症，减少肌损伤，同时疾病后期肌酸激酶下降后可尽量缩短大剂量激素使用疗程，同时辅以康复/支持治疗，以改善预后。

### 3.6 神经系统毒性

关于神经系统毒性，指南将其分为不同类型进行描述和推荐，总结如下：（1）重症肌无力：分级从2级开始，3度-4度毒性永久停止ICIs，住院治疗，甲基泼尼松龙起始量为1 mg/kg/d-2 mg/kg/d，根据病情调整剂量，但如何调整尚无详细介绍。避免使用可能加重肌无力的药物（如β-受体阻滞剂、含镁离子药物、喹诺酮类、氨基糖苷类及大环内酯类抗生素等）。治疗效果不佳时免疫球蛋白0.4 g/kg/d或者血置换，连续5 d。注意肺功能、神经系统症状。（2）格兰巴雷综合症：格兰巴雷综合症分级从2级开始，均需永久停止ICIs，住院或入住ICU监护病房，密切监测神经系统症状和呼吸功能，请神内科会诊。建议免疫球蛋白，0.4 g/kg/d，或者血浆置换，连续5 d。对疼痛患者，给予非阿片类药物治疗疼痛。也可试验性应用甲基泼尼松龙，2 mg/kg/d-4 mg/kg/d，随后逐渐缓慢减量；也有推荐使用甲基泼尼松龙，1 g/d，连续5 d，与免疫球蛋白或血浆置换联合应用；也有推荐先行免疫球蛋白或血浆置换治疗，类固醇激素逐渐增量，具体未详细介绍。（3）无菌性脑膜炎：3级-4级毒性时，永久停止ICIs，住院治疗。在脑脊液结果明确以前，考虑静脉注射无环鸟苷直至获得聚合酶链反应（polymerase chain reaction, PCR）结果，或静脉给予阿昔洛韦抗病毒治疗。除外细菌和病毒感染，密切监控而不使用类固醇激素。如果出现中重度症状，试验性应用甲基泼尼松龙1 mg/kg/d-2 mg/kg/d治疗脑炎。如诊断明确甲基泼尼松龙1 mg/kg/d-2 mg/kg/d，如果症状严重或者出现寡克隆带，给予甲基泼尼松龙，1 g/d，连续3 d-5 d，即类固醇激素增量。同时给予免疫球蛋白，0.4 g/kg/d，连续5 d。如果病情进展或出现自身免疫性脑病抗体，给予抗CD20单抗，如利妥昔单抗，或者血浆置换。在脑脊液结果明确以前，给予经验性抗病毒（静脉给予阿昔洛韦或无环鸟苷）及抗生素治疗。（4）横断性脊髓炎：永久停止ICIs，请神经内科会诊，给予甲基泼尼松龙2 mg/kg/d，根据病情，可给予冲击剂量甲基泼尼松龙1 g/d，连续3 d-5 d，免疫球蛋白0.4 g/kg/d，连续5天，或者血浆置换。

而SITC的指南则将神经系统毒性分为：中枢性损伤——脑病/脑白质病/可逆性后部白质脑病综合征（posterior reversible encephalopathy syndrome, PRES），建议3级-4级毒性永久停用ICIs，类固醇激素的起始剂量为1 mg/kg/d-2 mg/kg/d，并预防性使用抗生素3 d无好转准备透析；以及外周性损伤——外周运动和感觉神经病：3级毒性既为危重，永久停用ICIs，类固醇激素起始剂量为1 mg/kg/d-2 mg/kg/d，并预防性使用抗生素。

本文建议，对于以上神经系统irAE，类固醇激素治疗的同时，建议用免疫球蛋白或血浆置换联合应用，并且可按疗程反复使用，即21 d一次，共3次-4次的应用。免疫抑制剂方面，指南除了对于无菌性脑膜炎有给予抗CD20单抗的推荐外，其余均未推荐免疫抑制剂（生物类和非生物类）治疗。而神经系统疾病中，亚急性和慢性炎症性脱髓鞘性多发性神经根神经炎、重症肌无力、脑炎、无菌性脑膜炎等疾病均有从类固醇激素以外的免疫抑制剂治疗中获益的成功报道，如亚急性和慢性炎症性脱髓鞘性多发性神经根神经炎应用抗IL-1受体抑制剂治疗，重症肌无力应用抗IL-1受体抑制剂、抗IL-6受体抑制剂和抗CD20单抗治疗，脑炎和无菌性脑膜炎应用抗IL-1受体抑制剂和抗CD20单抗治疗等^[14-16]^。因此在危重的神经毒性中可探索特异性免疫抑制剂的治疗。

### 3.7 血液毒性

#### 3.7.1 贫血

包括自身免疫性溶血性贫血和再生障碍性贫血ESMO和NCCN/ASCO未涉及自身免疫性溶血性贫血，SITC提及病名但无处理意见，只有CSCO指南有较详细的描述：建议完善血常规、网织红细胞计数、大小便常规、外周血涂片、乳酸脱氢酶、直接和间接胆红素、叶酸、维生素B12、铁蛋白、血清铁、珠蛋白、骨髓象、Coombs直接间接试验，阵发性夜间血红蛋白尿等检查；排除药物、昆虫、蛇咬伤、细菌、病毒感染等导致的溶血性贫血；建议永久停用ICIs，泼尼松1 mg/kg/d-2 mg/kg/d，请血液科会诊，如无效或恶化，给予免疫抑制剂，如抗CD20单抗利妥昔单抗、免疫球蛋白、环孢素和麦考酚酯等；可根据指南输红细胞纠正贫血，补充叶酸1 mg/d。

关于再生障碍性贫血也只有CSCO指南进行了较详细的描述：建议完善血常规、网织红细胞、骨髓象、维生素B12、叶酸、铁蛋白、血清铁、肝肾功能、病毒等检查，并排除药物、辐射、毒素、病毒感染等导致的再生障碍性贫血以明确诊断；建议暂停ICIs，每天密切随访，血液科会诊；建议造血生长因子，ATG+环孢素、环磷酰胺等治疗；建议对难治性患者给予艾曲波帕和支持治疗；可输血，但所有的血液制品应接受照射和过滤。

我们建议，对于类固醇激素的使用与否应给予说明。目前的指南建议均遵循再生障碍性贫血的一般治疗原则，这些治疗是否适用于ICI引起的贫血，还有待于临床实践中的进一步探索。

#### 3.7.2 免疫性血小板减少症

ESMO和NCCN/ASCO未涉及免疫性血小板减少症，SITC提及病名但无处理建议，只有CSCO指南有较详细的描述：建议检测血常规、骨髓象、自身抗体、血小板抗体、病毒或细菌检测等，同时需排除药物、其他自身免疫性疾病、病毒感染引起的血小板减少症、再生障碍性贫血等疾病以明确诊断；3级以上毒性时暂停ICIs，密切随访及治疗，如果恢复到1级毒性以下可继续治疗；可予以泼尼松1 mg/kg/d-2 mg/kg/d口服，如果无缓解或者恶化，继续使用泼尼松，并联合静脉输注免疫球蛋白1 g/kg，并根据需要重复使用；也可考虑使用抗CD20单抗利妥昔单抗、血小板生成素受体激动剂艾曲波帕^[17]^。

我们认为，对CSCO指南中关于“泼尼松1 mg/kg/d-2 mg/kg/d口服，如果无缓解或者恶化，继续使用泼尼松，并联合静脉输注免疫球蛋白1 g/kg，并根据需要重复使用”的建议临床上应谨慎应用，对激素效果差者应早期考虑血小板生成素受体激动剂艾曲波帕、抗CD20单抗利妥昔单抗等药物的使用。

#### 3.7.3 获得性血友病

关于获得性血友病同样仅于CSCO指南中有较详细描述：建议检测血常规、纤维蛋白原、凝血酶原时间（prothrombin time, PT）、活化部分凝血酶时间测定（activated partial thrombin time determination, APTT）、APTT纠正实验、凝血因子定量、Bethesda凝血因子抑制物测定，并采用磁共振成像、计算机断层扫描或超声对出血进行定位、定量和连续监测以明确诊断获得性血友病；3级以上毒性时永久停止使用ICIs，血液科会诊；根据Bethesda法检测抑制物的表达水平选择凝血因子替代治疗，给予1 mg/kg/d泼尼松±抗CD20单抗利妥昔单抗1 mg/kg/d-2 mg/kg/d、环磷酰胺和输血。如果继续恶化给予环孢素或其他免疫抑制剂。

本文建议还应关注免疫性中性粒细胞减少症的发生，如有发生推荐联合静脉输注免疫球蛋白1 g/kg，按疗程使用。另外关于ICI治疗相关的嗜酸性粒细胞增多症、噬血细胞性淋巴组织细胞增多症也有报道^[18]^。

### 3.8 肾脏毒性

指南建议对于3级肾脏毒性即永久停用ICIs，每24 h监测肌酐和尿蛋白，予泼尼松/甲基泼尼松龙（1 mg/kg/d-2 mg/kg/d），若1周后仍 > 2级可考虑加用硫唑嘌呤/环磷酰胺/环孢霉素/英夫利西单抗/麦考酚酸酯。4度毒性危及生命者需急诊透析。需除外脱水、近期静脉造影、尿路感染、药物、低血压或高血压等原因，尽早考虑肾活检。

我们建议还应注意辨别急进性肾小球肾炎、免疫介导的肾炎、免疫相关肾功能衰竭、肾病综合征等不同类型肾损伤并进行相应治疗。在鉴别诊断时应除外膀胱受累。肾脏损伤的发生率较低，尚无更多的病例报道和积累，免疫抑制剂方面除英夫利西单抗尚无其他推荐。

### 3.9 心脏毒性

3度-4度心肌毒性需立即请心内科会诊，完善心电图检查、心肌损伤标志物（肌酸激酶和肌钙蛋白）、炎性标志物[红细胞沉降率、C反应蛋白、白细胞等]、心脏彩超和/或心肌增强MRI检查，心电监护。永久停用ICIs。给予甲基泼尼松龙冲击，1 g/d，持续3 d-5 d，NCCN/ASCO和CSCO指南推荐治疗至心功能恢复基线后，在4周-6周内逐渐减量。激素治疗24 h无改善时考虑加用ATG、英夫利昔单抗（但应注意的是，英夫利昔单抗与心力衰竭有关，对中-重度心力衰竭患者禁用大剂量英夫利昔单抗）。针对心肌炎或心包炎的治疗（抗病毒治疗、免疫球蛋白或透析治疗）。

我们建议，因心肌炎多发生在接受第1周期-2周期ICI治疗后，故临床医生在患者开始ICI治疗时即应提高警惕，应连续复查ECG、检测心肌酶谱的变化，同时加强患者宣教，这对于早期识别心肌炎至关重要。而良好的转归与早期发现和治疗直接有关^[19]^。致死性心脏炎中以心律失常、房室传导阻滞引起的死亡率最高，其中36%的患者可发生完全性房室传导阻滞，这些患者中有60%可出现死亡，而其中21%的患者出现心律失常时肌钙蛋白/肌酸激酶水平并未明显升高，这种情况下心脏起搏器的植入和全面的心内科专科治疗对于改善预后最为重要。部分心肌炎的患者尽管在激素冲击治疗后心肌酶稳步下降，后续仍可出现恶性心律失常导致死亡，心肌活检也证实心肌内存在未控制的炎症^[20]^，对于这部分患者，新的特异性免疫抑制药物治疗可能是成功的关键。我们推荐对于重症心肌炎病人可在激素基础上使用抗IL-1受体抑制剂和抗IL-6受体单抗。抗IL-6单抗在大动脉炎的治疗中有推荐，在继发于成人Still病的爆发性心肌炎中也有成功的报道^[21]^，而其安全性也已经得到证实。另外，文献也有报道用抗CD52单抗-阿仑单抗清除外周血T细胞而成功治愈难治性心肌炎的病例^[22]^，以及用CTLA-4激动剂阿巴他西普（每2周500 mg，共5剂），竞争性结合CD80/CD86抑制T细胞的辅刺激信号通路治疗难治性心肌炎^[23]^，以及用抗胸腺细胞球蛋白ATG（500 mg/d, 5 d）治疗成功的个案^[20]^。然而，对于这些药物，其安全性是限制使用的主要因素，尤其是继发感染的风险，毕竟，免疫抑制后继发感染也是重症心肌炎的常见死亡原因。

### 3.10 眼毒性

（1）葡萄膜炎：后葡萄膜炎或全葡萄膜炎属3度毒性。根据发病的严重程度、前期对ICIs治疗的获益以及对糖皮质激素治疗的反应，谨慎选择少部分患者恢复ICIs治疗。而4度毒性开始激素治疗前请眼科会诊，根据建议使用局部或全身糖皮质激素治疗，但应永久停用ICIs。（2）巩膜炎：3度-4度毒性原则上永久停用ICIs。3度根据发病的严重程度、前期对ICIs治疗的获益以及对糖皮质激素治疗的反应，谨慎选择少部分患者恢复ICIs治疗。3度-4度毒性均应于开始激素治疗前请眼科会诊，根据建议使用局部或全身糖皮质激素治疗。

我们建议还应关注ICIs治疗引起眼眶炎、巩膜外层炎、眼睑炎、视神经水肿、溃疡性结膜炎及伴有黄斑，另外也有关于浆液性视网膜剥脱的Vogt-Koyanagi-Harada综合征的报道^[24]^。其他报道的眼部异常还包括黄斑水肿、视网膜病变、眼肌炎、眼眶炎症、角膜穿孔、眼内炎症等。关于类固醇激素以外免疫抑制剂的应用目前很少有报道。但是从发病机理和脏器特点上看，免疫抑制剂的应用仍然有前景。

综上五大irAE相关指南/共识的指导意见，我们发现，由于缺乏大样本的临床研究及经验，指南关于危重/难治性irAE的这部分内容均不够系统、全面和详细，缺乏支持依据，甚至有照搬原发性自身免疫病的情况。而以上我们的关于指南以外的补充或建议有些尚不成熟，但可作为未来在irAE管理方面探索的方向。

## 4 类固醇激素以外可用于治疗irAE的药物应用的初步探讨

目前irAE的治疗仍以类固醇激素为基础，而难点是难治性irAE的治疗选择。而新型、罕见的，有时甚至是致命的irAEs还在出现。对于这一类病因明确、机制明确的特殊疾病，治疗上如何快速的抑制免疫、控制免疫抑制剂本身带来的副作用，同时选择器官特异性的治疗可能是将来提高成功率的关键。为此，新型的生物制剂以及针对细胞因子的靶向药物的应用值得期待。表 1所示为指南中涉及的一些细胞因子靶向免疫抑制剂，及其常规使用方法（表 1）。

**1:** 推荐用于irAEs治疗的相关细胞因子靶向免疫抑制剂 Cytokine-targeted immunosuppressive agents that were recommended

	Properties	Generic name	Usage and dosage
1	IL-6 receptor antagonist	Tocilizumab	8 mg/kg，intravenous, once a month, or 162 mg, subcutaneous injection, once a week
2	Immunoglubin	Immunoglubin	400 mg/kg/d, intravenous for 5 d,
3	Anti-CD20 antibody	Rituximab	1 g, once every 14 d, for two cycles, or 375 mg/m^2^, once a week, for 4 cycles
		Ofatumumab	300 mg for d1, 1, 000 mg for d2
		Obinutuzumab	1, 000 mg for d1
		Ocrelizumab	300 mg for d1, 300 mg for d4
4	Anti-TNFα antibody	Infliximab	5 mg/kg, once every 2 weeks
		Adalimimab	40 mg, once every 2 weeks
		Golimumab	50 mg, once a month
		Certolizumab	400 mg, once a month
		Etanercept	50 mg, once a week
5	Anti-integrin a4 antibody	Natalizumab	300 mg, once a month
		Vedolizumab	300 mg, once a month
6	Thrombopoietin receptor agonist	Eltrombopag	50 mg/d, Oral
7	Anti-thymocyte globulin	ATG	500 mg/d, d1-5
6	Janus kinase inhibitor	Tofacitinib	2 mg, *bid*, oral
irAEs: immune-related adverse effects.			

部分细胞因子靶向免疫抑制剂在irAE中的使用已经被证实或正在被推广，如抗TNFα单抗的使用，在心肌炎、结肠炎等均有推荐，而进一步明确其适用范围及副作用是关键。抗IL-6的拮抗剂在CAR-T细胞引起的细胞因子释放综合征中有良好的疗效，在危重及难治性irAE的治疗中也已经显示出一定的作用^[25]^。在急性炎症期不断分泌的细胞因子是免疫损伤及免疫反应级联的关键，特别是IL-1、IL-6和TNFα，阻断这些细胞因子会减少其对辅助性T细胞、B细胞、自然杀伤细胞、巨噬细胞、浆细胞和造血干细胞的刺激作用和对内皮激活作用。这可能比经典倡导的抗TNFα策略更有效。因为IL-6和IL-1的促肿瘤和促转移活性，通过优先使用抗IL-6单抗或抗IL-1单抗来达到“关闭”策略，还有可能实现额外的抗肿瘤优势。在ICIs诱导的脑炎病例中，抗IL-1疗法也可以是有用的辅助治疗，其中炎症反应主要由IL-1增加驱动。抗B细胞抗体可能有助于ICI的神经或血液学并发症以及ICI诱导的结缔组织疾病，严重的Stevens-Johnson综合征和血管炎相关的irAEs。另外，IL-12/23抑制剂可以通过削弱IL-23对TNFα分泌的正刺激作用来抑制急性炎症期，这可以在抗TNFα药物难以治疗的irAE病例中体现。在抗IL-6治疗失败后或抗TNFα治疗失败的难治性银屑病样反应的情况下，抗IL-17策略可能是一个很好的选择。

而进一步探索危重及难治性irAE的治疗，不仅包括激素耐药后的药物选择，还包括是否可在疾病初期联系细胞因子靶向的免疫抑制剂治疗，以尽早抑制疾病初期快速进展的病理损伤过程，并缩短后期免疫高度抑制的时期以减少感染风险，最终达到成功治疗的目的^[9]^。

## 5 总结

总之，类固醇激素可以治疗大部分irAE，尤其是轻型的irAE，但是确实存在有部分类固醇耐药或单纯类固醇激素不能应对的难治性irAE，需要其他的免疫抑制剂的应用，尤其是特异性的免疫抑制剂的应用。

超大剂量的类固醇激素只有在少数情况下推荐。反复使用超大剂量激素需要避免（除免疫性血小板减少症）。

在危重症的治疗中，临床疗效的观察方式包括症状、体征、实验室检查和影像及功能方面的评估，观察的窗口期为24 h、48 h和72 h不等，如果病情无好转应当立即进行其他的干预治疗。

对于特定的irAE，专科的支持治疗，包括外科介入治疗都很重要，但是必须结合晚期肿瘤患者的特点，及其irAE的病程特点予以综合考虑，在处理irAE的同时需高度关注恶性肿瘤的行为。
